# Genome Sequences of Two Choline-Utilizing Methanogenic Archaea, *Methanococcoides* spp., Isolated from Marine Sediments

**DOI:** 10.1128/MRA.00342-19

**Published:** 2019-05-02

**Authors:** Gordon Webster, Alex J. Mullins, Andrew J. Watkins, Edward Cunningham-Oakes, Andrew J. Weightman, Eshwar Mahenthiralingam, Henrik Sass

**Affiliations:** a Microbiomes, Microbes and Informatics Group, Organisms and Environment Division, School of Biosciences, Cardiff University, Cardiff, Wales, United Kingdom; b School of Earth and Ocean Sciences, Cardiff University, Cardiff, Wales, United Kingdom; Georgia Institute of Technology

## Abstract

The genomes of two Methanococcoides spp. that were isolated from marine sediments and are capable of carrying out methanogenesis from choline and other methylotrophic substrates were sequenced. The average nucleotide identity and *in silico* DNA-DNA hybridization analyses demonstrate that they represent species different from those previously described.

## ANNOUNCEMENT

The genus Methanococcoides comprises four described and characterized species, M. methylutens (1), M. burtonii (2), M. alaskense (3), and M. vulcani (4), as well as several other strains isolated from marine and mangrove sediments (5–7). To date, all Methanococcoides species are obligate methylotrophs able to utilize a range of methylated compounds for methanogenesis and belong to the diverse methanogen family *Methanosarcinaceae* (8).

Two Methanococcoides strains (with >98% 16S rRNA gene similarity to *M. methylutens*) (5) were isolated from sediments of Aarhus Bay, Baltic Sea (AM1), and Napoli Mud Volcano, eastern Mediterranean Sea (NM1), in artificial seawater (ASW) supplemented with methylamine, using deep-agar shake tubes and a dilution-to-extinction series with antibiotics to inhibit bacterial growth at 25°C (5).

For genome sequencing, each strain was grown in 2 × 10 ml ASW with 10 mM trimethylamine in tubes fitted with rubber stoppers and caps. Cells collected by centrifugation were washed with ASW and transferred to Lysing Matrix E tubes, and DNA was extracted using the FastDNA Spin kit (MP Biomedicals) (9). Sequencing was performed on an Illumina NextSeq 500 platform using a Nextera XT DNA library preparation kit. For each genome, 0.5 to 1.0 million 2 × 150-bp paired-end reads were generated. Illumina adaptors were trimmed with Trim Galore version 0.4.2, quality was assessed using FastQC version 0.10.1, and contigs were assembled *de novo* using SPAdes version 3.9.1. The genome assemblies had 30× (AM1) and 64× (NM1) coverage. The genome metrics for the two assemblies are as follows: for AM1, 2.48 Mbp, 42.66% G+C content, an *N*_50_ value of 433,016 bp, and 46 contigs; and for NM1, 2.34 Mbp, 43.18% G+C content, an *N*_50_ value of 241,508 bp, and 45 contigs. Both genome sizes are close to those reported for other *Methanococcoides* species (Table 1). A total of 2,445 (AM1) and 2,292 (NM1) coding DNA sequences (CDS) were identiﬁed using the Prokka version 1.12-beta genome annotation tool (8). The AM1 and NM1 genomes contained 6 and 7 rRNAs, 44 and 46 tRNAs, and 3 and 2 predicted CRISPR regions and several cytochromes, respectively. The entire operon encoding methyl coenzyme M reductase (Mcr) and genes for methanogenesis (*fmd*, *ftr*, *mch*, *mtd*, *mer*, *mtrABCDEFGH*, and *hdrABCDE*) were present. The genomes contained evidence of monomethylamine, dimethylamine, trimethylamine, and methanol metabolism, with genes encoding methanol-corrinoid protein comethyltransferase (Mta), monomethylamine methyltransferase (Mtm), dimethylamine methyltransferase (Mtb), and trimethylamine methyltransferase (Mtt), along with the corresponding corrinoid protein genes. Both strains had genes encoding methylsulfide methyltransferase-associated sensors predicted to be involved in two-component regulation systems (10).

**TABLE 1:** Pairwise ANI and *in silico* DDH between the novel strains and other *Methanococcoides* species and their respective genome sizes

Strain genome (description and source or accession no.)	Genome size (Mbp)	Pairwise ANI (%) with genome of^*a*^:		Pairwise DDH (%) with genome of^*b*^:	
		AM1	NM1	AM1	NM1
AM1	2.48		90.4		39.0
NM1	2.34	90.4		39.0	
*M. methylutens* DSM 2657^T^ (JRHO00000000)	2.51	90.1	89.7	37.7	37.1
*M. burtonii* DSM 6242^T^ (CP000300)	2.58	84.5	85.1	21.0	21.6
*M. vulcani* SLH33^T^ (FOHQ00000000)	2.31	90.6	95.8	39.8	64.7
*M. methylutens* MM1 (CP009518)	2.39	85.6	85.5	26.2	26.7
*M. methylutens* DSM 2657^T^ (this study)	2.50	90.2	89.8	37.6	37.1
*M. vulcani* SLH33^T^ (this study)	2.32	90.6	95.9	39.7	64.8
*Methanococcoides* sp. strain EBM-47 (anaerobic digester metagenome; MPPA00000000)	2.18	83.7	83.3	15.2	15.8

a Average nucleotide identity (ANI) values of <95% indicate different species.

b *In silico* DNA-DNA hybridization (DDH) values of <70% indicate different species.

*Methanococcoides* strains were subject to average nucleotide identity (ANI) analysis using PyANI (https://github.com/widdowquinn/pyani) and *in silico* DNA-DNA hybridization (DDH) with the Genome-to-Genome Distance Calculator (GGDC) 2.1 (11). The AM1 genome possessed ANI values below 95% compared to previously recognized species, while the NM1 genome identity was just above 95% compared to *M. vulcani* (Table 1). An ANI value of <95% has been proposed for species delineation (12). The ANI comparisons indicated that AM1 represents a novel species, while NM1 is closely related to *M. vulcani*. However, analysis by *in silico* DDH produced values for both strains below the 70% species threshold compared with genomes from described *Methanococcoides* species, including NM1 with *M. vulcani* (Table 1). The ANI and DDH values together suggested that AM1 and NM1 represent two phylogenetically different species of *Methanococcoides* from those described previously and warrant further characterization.

### Data availability.

The genome sequences and Illumina raw sequence reads have been deposited at the European Nucleotide Archive (ENA) under the ENA project/study number PRJEB31721. The accession numbers for the genomes are CAAGSW010000000 for AM1 and CAAGTW010000000 for NM1. The genomes of *M. methylutens* DSM 2657 and *M. vulcani* SLH33 were also resequenced and submitted under accession numbers CAAGSM010000000 and CAAGSJ010000000, respectively.
